# Variability in Carob Fruit Composition: Geographic and Maturity Stage Effects on Physical and Biochemical Properties

**DOI:** 10.1155/sci5/6783176

**Published:** 2026-07-31

**Authors:** Salah Laaraj, Chaimae El-Rhouttais, Hicham Ouhakki, Aziz Tikent, Sofia Zazouli, Atman Adiba, Mohamed Kouighat, Abdellatif Boutagayout, Anas Hamdani, Mohamed Lamsiah, Ayour Jamal, Mohammed Mitache, Souad Salmaoui, Kaoutar Elfazazi

**Affiliations:** ^1^ Regional Center of Agricultural Research of Tadla, National Institute of Agricultural Research (INRA), Rabat 10090, Morocco, inra.org.ma; ^2^ Environmental, Ecological, and Agro-Industrial Engineering Laboratory, LGEEAI, Faculty of Science and Technology (FST), Sultan Moulay Slimane University (USMS), Beni Mellal, Morocco, fst-usmba.ac.ma; ^3^ Laboratory of Natural Resources and Sustainable Development, Department of Biology, Faculty of Science, University Ibn Tofail, BP, 133-14000, Kenitra, Morocco, uiz.ac.ma; ^4^ Laboratory of Agricultural Production Improvement, Biotechnology and Environment (LAPABE), Faculty of Sciences, Mohammed Premier University, Oujda, Morocco, ump.ma; ^5^ Molecular Chemistry, Materials and Catalysis Laboratory, Faculty of Sciences and Techniques, Sultan Moulay Slimane University, B.P. 523, 23000, Beni-Mellal, Morocco, universitesms.com; ^6^ Research Unit on Environment and Sustainable Management of Natural Resources, Regional Center of Agricultural Research of Settat, National Institute of Agricultural Research, Avenue Ennasr BP 415, Rabat Principale 10090, Morocco, inra.org.ma; ^7^ The Environment and Soil Microbiology Unit, Faculty of Sciences, Moulay Ismail University, P.O. Box 11201, 50000, Zitoune, Meknes, Morocco, umi.ac.ma; ^8^ Research Unit on Environment and Conservation of Natural Resources, Regional Center of Agricultural Research of Rabat, National Institute of Agricultural Research, Avenue Ennasr BP 415 Rabat Principale, 10090, Rabat, Morocco, inra.org.ma; ^9^ TEDAEEP Research Team, Department of Life Sciences, Polydisciplinary Faculty of Larache, Abdelmalek Essaadi University, 745 BP, 92004, Larache, Morocco, uae.ma; ^10^ Bioprocess and Environment Team, LASIME Laboratory, Agadir Superior School of Technology, Ibn Zohr University, BP 33/S, Agadir, Morocco, uiz.ac.ma; ^11^ Research Unit of Plant Breeding and Genetic Resources Conservation, Regional Center of Agricultural Research of Settat, National Institute of Agricultural Research, Settat, Morocco, inra.org.ma

**Keywords:** bioactive compounds antioxidant activity, carob pods, *Ceratonia siliqua* L., geographic origin, maturity stage

## Abstract

This study investigates the impact of geographic origin and maturity stage on the morphological and biochemical characteristics of immature carob (*Ceratonia siliqua* L.) fruits collected from three distinct locations in central Morocco. A total of 300 trees were sampled across seven phenological stages (*T*_0_–*T*_7_), and various parameters were assessed, including fruit morphology, colorimetric indices, sugar content, total polyphenols, flavonoids, tannins, and antioxidant activities (DPPH, ABTS, and FRAP). Two‐way ANOVA revealed that these attributes were significantly influenced by the site, the maturity stage, and their interaction. Morphological characteristics increased significantly throughout fruit maturation, with the most pronounced changes occurring during the advanced developmental stages (*T*_5_–*T*_7_). Pod length, width, and thickness exhibited a progressive upward trend, reaching their highest values at late maturity stages. In particular, pod length increased markedly from 4.27 ± 0.45 cm at *S*_2_–*T*_0_ to 21.43 ± 2.65 cm at *S*_3_–*T*_5_, corresponding to an approximately fivefold increase during fruit development, thereby highlighting the substantial morphological changes associated with pod maturation. Bioactive compounds showed stage‐dependent variations, with the highest levels generally recorded at intermediate maturity stages (*T*_1_–*T*_2_). Total phenolic content peaked at *T*_2_, reaching 3342.38 ± 377.96 mg GAE/100 g, while total flavonoid and condensed tannin contents attained maximum values of 1335.42 ± 187.95 mg QE/100 g and 1149.21 ± 55.26 mg CE/100 g, respectively. Principal component analysis accounted for 83.64% of the total variability, highlighting clear groupings based on the developmental stage and site‐specific effects. These findings demonstrate that the quality of immature carob fruits is strongly dependent on both environmental and developmental factors. The results provide a valuable basis for the targeted use of carob fruits in food, pharmaceutical, and nutraceutical applications, optimizing their nutritional and functional potential.

## 1. Introduction

The carob tree (*Ceratonia siliqua* L.), a species adapted to the Mediterranean basin, is also grown in temperate and subtropical regions. This combination of drought tolerance, ability to grow growth under low fertility conditions, resistance to environmental stress, and its ecological, nutritional, and medicinal importance makes it a highly valued plant [1]. The carob tree, with its deep root system, contributes to the stabilization of soil defending against erosion and desertification processes [2–4].

The whole carob fruit is among the various components that have received increasing attention within food science [5] due to its valuable compositional profile. The pulp, which represents about 90% of the weight of the pod, is rich in sugars (48%–56%) and dietary fiber (27%–50%), and low in protein and lipids compared to carob seeds [6–8]. Substantial amounts of polyphenols and minerals are also present to substantiate its use in functional foods and nutraceuticals [9–11]. The functional properties of carob pulp have also made it well accepted in food and pharmaceutical applications as a natural sweetener, cocoa substitute, and source of bioactive compounds [12]. However, the content of carob pods varies considerably among trees, variety, geographical origin, and cultivation conditions of environmental factors and stage of maturity [13]. The concentrations of polyphenolic compounds, such as tannins and flavonoids, are particularly high in immature pods and then decrease with ripening, while the sugar content gradually increases [14, 15].

In the last few years, the potential therapeutic value of immature carob fruits has become more popular. Some of the important bioactive compounds such as gallic acid and D‐pinitol are known to exert a multitude of biological activities, which include antioxidant, antihyperglycemic, hypolipidemic, and anti‐inflammatory activities [16–19]. These bioactive metabolites are especially important in the prevention and treatment of metabolic diseases such as diabetes, cardiovascular diseases, and some cancers [5, 20, 21].

Immature pods of *C. siliqua* have comparatively low quantities of soluble sugars, since carbon allocation is primarily directed towards active growth, cellular division, and structural development instead of the formation of storage metabolites. At this stage, carbohydrate metabolism is mostly governed by biosynthetic mechanisms that facilitate pod expansion, resulting in limited sucrose accumulation. Conversely, maturity entails a dramatic metabolic reprogramming, resulting in a substantial elevation of soluble sugars, especially sucrose, which emerges as the principal carbohydrate in fully formed pods. This general pattern has been extensively documented in carob throughout Mediterranean populations, including Morocco, Spain, and Portugal, where fruit maturity is consistently linked to a transition from low‐sugar immature tissues to high‐sucrose mature pods, attributable to alterations in carbon partitioning and enzymatic regulation of carbohydrate metabolism [22–24].

While the results are encouraging, there is a paucity of research examining the influence of environmental factors and developmental stages during the immature phase. It is imperative to comprehend these factors in order to facilitate the optimal utilization of carob fruits in both functional and therapeutic applications. This study aims to evaluate differences in the physicochemical properties of immature *C. siliqua L.* fruits according to geographical origin and phenological stage in central Morocco. The study will focus on the specific morphological traits, sugar content, and phenolic compound concentrations, namely, polyphenols, flavonoids, and condensed tannins, as well as antioxidant activity via DPPH, ABTS, and ferric reducing antioxidant power (FRAP). This study presents a valuable set of findings that offer a comprehensive understanding of the variation in the content of immature carob pods according to the growth stage and geographical location. The results provide a scientifically sound framework for determining the significant content of phenolic compounds in the first place, thus contributing to sustainable solutions for the valorization of carob products at different stages in potential applications in the food, pharmaceutical, and nutraceutical industries.

## 2. Materials and Methods

### 2.1. Plant Materials

Carob pods at the immature stage were systematically collected from three distinct stations. The Bin El Ouidane commune (Station 1 (*S*_1_), coordinates: 32°08′41.1″N 6°30′04.8″W), the commune of Timoulilt (Station 2 (*S*_2_), coordinates: 32°11′52.9″N 6°19′52.2″W), and the commune of Ouaouizeght (Station 3 (*S*_3_), coordinates: 32°12′24.9″N 6°23′30.8″W). These sites are located in the Beni Mellal–Khenifra region of central Morocco, and sampling was conducted during the period from April to June 2023. The primary objective was to track the developmental stages of the pods before reaching full maturity.

The immature stage harvest was conducted according to seven distinct developmental stages (*T*_1_, *T*_2_, *T*_3_, *T*_4_, *T*_5_, *T*_6_, and *T*_7_), which were established based on visible morphological and physiological criteria commonly used in fruit developmental studies. Stage differentiation was performed according to pod dimensions, color evolution, texture, and progression of fruit growth throughout maturation. A total of 30 trees were selected, with ten trees from each of the three collection areas, resulting in a total of 50 pods harvested per tree. A systematic sampling approach was employed, where an equal number of pods were collected from each of the four cardinal directions (north, south, east, and west) and from different parts of the tree. This ensured a comprehensive and representative sampling. Only pods that were observed healthy, free from disease symptoms, and without physical defects were selected to ensure the quality and integrity of the study.

### 2.2. Pomological Trait Measurement

Pomological analyses were carried out on a randomly selected sample of 15 pods, with three replicates per sample, corresponding to five pods per replicate. The parameters assessed included pod length, width, thickness, and chord. Additionally, the pod weight, number of seeds, seed weight, and seed yield percentage were determined.

### 2.3. Color Attributes

The color of unripe carob pods was measured in CIELAB coordinates (*L* ^∗^, *a* ^∗^, *b* ^∗^) using a Minolta Chroma Meter CR‐400 (Minolta Corp., Osaka, Japan) [25]. These color measurements were used to track changes in the external appearance of the pods throughout their development. The measurements were taken from three predefined surface areas on each pod. To further analyze the color, the chroma (*C* ^∗^) and hue angle (*h*°) were calculated using the following equations:

(1) C∗=a∗2+b∗212/,

(2) h°=tan−1ba.

### 2.4. Preparation of Immature Carob Juice

Upon arrival at the laboratory, the immature carob pod was crushed using an electric juice extractor (Mellerware, South Africa). The harvested juice underwent centrifugation at 4500 rpm (2150 × *g*) for 15 min using a Sigma 2‐16P centrifuge (Sigma Laborzentrifugen GmbH, Osterode am Harz, Germany). The supernatant was preserved in opaque containers in the refrigerator to prevent oxidation. The carob juice was utilized for compositional analysis.

### 2.5. pH and Total Soluble Solids

The pH of the juice was measured at room temperature with a pH meter (Thermo Orien 3 Star, USA). TSSs were determined using a digital refractometer (Mettler‐Toledo GmbH, Switzerland).

### 2.6. Total Sugar Content (TSC)

The quantification of total sugars was carried out following the method outlined by Ref. [26], with minor modifications. Initially, 0.5 mL of juice extracted from the immature pods was mixed with 2.5 mL of sulfuric acid. Subsequently, 0.5 mL of phenol (5% w/v) was added. After a period 40 min of dark incubation, the absorbance was measured at 488 nm using a Shimadzu UV‐2401 PC UV spectrophotometer.

### 2.7. Total Phenolic Content (TPC)

Following a slightly modified version of the Folin–Ciocalteu colorimetric method [5], a mixture consisting of 100 μL of juice, 0.5 mL of Folin–Ciocalteu reagent, and 0.4 mL of a 7.5% sodium carbonate solution was incubated for 75 min at ambient temperature. After incubation, the absorbance of the mixture was measured at 760 nm using a Shimadzu UV‐2401 PC spectrophotometer. The TPC was determined using a standard curve and expressed as mg of gallic acid per 100 g fresh weight (FW).

### 2.8. Total Flavonoid Content (TFC)

The flavonoid content was determined using the AlCl_3_ method [27] with slight modifications. A 0.5‐mL sample of juice was mixed with 1500 μL of 95% ethanol, 0.5 mL of 2% (w/v) AlCl_3_ solution, 100 μL of 1 M sodium acetate, and 2.8 mL of distilled water. For 30 min, the mixture is incubated at ambient temperature in dark settings. Replace the juice with distilled water to make the blank, and then measure the absorbance at 415 nm shaken using a Shimadzu UV‐2401 PC spectrophotometer. The flavonoid content was calculated and expressed as milligrams of quercetin equivalent (mg QE) per 100 g FW.

### 2.9. Total Condensed Tannins (TCTs)

Condensed tannins in the carob juice were determined by adapting the method outlined by Ref. [28]. A 200‐μL sample of juice was mixed with 1.5 mL of 4% methanol vanillin solution, followed by the addition of 750 μL of concentrated hydrochloric acid (37%). The mixture was incubated in the dark for approximately ten minutes. Afterward, the absorbance was measured at 500 nm. The concentration of condensed tannins was calculated and expressed as catechin equivalents per 100 g FW.

### 2.10. Antioxidant Activity

The antioxidant capacity of unripe carob pods was evaluated using three assays: DPPH, ABTS, and FRAP.

#### 2.10.1. DPPH Assay

The DPPH assay was conducted to assess the antioxidant capacity of carob juice, following the method described by Ref. [29]. A 0.1‐mL sample of juice, diluted 1000‐fold with 60% ethanol, was mixed with 2 mL of DPPH solution (0.1 mM). The mixture was shaken and incubated for approximately 45 min. Absorbance was measured at 517 nm using a Shimadzu UV‐2410 PC spectrophotometer. A Trolox standard (1–5 mg/mL) was used (*R* ^2^ = 0.9706), and the free radical‐scavenging activity of the juice samples was expressed as milligrams of Trolox equivalents per 100 g FW.

#### 2.10.2. ABTS Assay

The ABTS assay was performed following the protocol of Ref. [30] to evaluate the antioxidant capacity. Initially, a 7 mM ABTS solution was mixed with 2.45 mM potassium persulfate and incubated in the dark at room temperature for 12–16 h. Prior to the experiment, the solution was diluted with ethanol to achieve an absorbance of 0.70 ± 0.02 at 734 nm. To assess the ABTS scavenging activity as mg Trolox equivalents per gram dry weight (mg TE/100 g FW), 1 mL of extract from each juice sample was mixed with 2 mL of the standard ABTS solution and left at ambient temperature for 30 min. The absorbance of the solution was then measured at 734 nm. Triplicate measurements were taken to ensure accuracy.

#### 2.10.3. FRAP Assay

The FRAP assay for determining the antioxidant potential of carob juice samples was conducted following the methodology of Ref. [31]. To prepare the FRAP reagent, 10 mmol/L TPTZ, 40 mmol/L FeCl_3_, and 0.3 M acetate buffer (pH = 3.6) were mixed in a 10:1:1 ratio. A 3‐mL aliquot of the freshly prepared FRAP reagent was combined with the juice sample, vortexed, and allowed to incubate for 30 min. Trolox standards ranging from 1 to 5 mg/mL (*R* ^2^ = 0.9706) were used, and the absorbance was measured at 593 nm. The results were expressed as milligrams of Trolox equivalents per 100 g FW.

### 2.11. Remote Sensing–Based Analysis of Vegetation Density and Water Content

#### 2.11.1. Normalized Difference Vegetation

The Normalized Difference Vegetation Index (NDVI) is a widely used spectral index for assessing the density and physiological vigor of vegetation. It is derived from the reflectance values in the near‐infrared (NIR) and red (red) bands according to the following formula:

(3) NDVI=NIR−redNIR+red.

NDVI values generally range from −1 to +1:

• Values close to 0 or negative indicate nonvegetated surfaces (bare soil, rock, or water).
• Values between 0.2 and 0.5 represent sparse to moderate vegetation.
• Values above 0.5 correspond to dense and vigorous vegetation.

#### 2.11.2. Normalized Difference Water Index (NDWI)

The NDWI is a spectral index sensitive to the water content of vegetation and soil. It is calculated using the green (green) and NIR bands according to the formula:

(4) NDWI=green−NIRgreen+NIR.

Positive NDWI values indicate high water content in the vegetation, while negative values reflect low water content, typically associated with water stress.

#### 2.11.3. NDWI Geo

The NDWI Geo, or Geometric NDWI, is a variant of the classical NDWI calculated using different spectral bands, typically NIR and short‐wave infrared (SWIR), following the formula:

(5) NDWIGeo=NIR−SWIRNIR+SWIR.

This index is more sensitive to internal water content in plant tissues and surface soil moisture. Positive values indicate better hydration of vegetation, while lower values suggest drier conditions.

### 2.12. Statistical Analysis

All statistical analyses were performed using R software (Version 4.4.3) (R Core Team, 2025). Descriptive statistics, including the minimum, maximum, mean, standard deviation (SD), and coefficient of variation (CV), were calculated for each variable across various stations and stages to provide a comprehensive summary of data distribution and variability.

Before conducting statistical analyses, the normality of variances was assessed employing the Shapiro–Wilk test (car package). In instances where a variable exhibited deviations from normality, suitable transformations, viz., logarithmic, square root, or exponential, were implemented to satisfy the requisite assumptions. To evaluate the effect of maturity (eight stages, from *T*_0_ to *T*_7_) on the physicochemical and biochemical properties of carob fruit across three distinct stations (*S*_1_, *S*_2_, and *S*_3_), a two‐way analysis of variance (ANOVA) was executed using the aov (…) function (stats package). When significant differences were detected, Tukey’s honest significant difference (HSD) test was utilized to determine specific stations and stages manifesting statistically significant variations. Different lowercase letters indicate significant differences among stages within the same station, while different uppercase letters indicate significant differences among stations within the same stage.

Pearson’s correlation analysis was employed to investigate the relationships among the measured traits using the cor function from the stats package. To analyze the patterns of variation and to identify the most influential traits contributing to the disparities among stations and stages, a principal component analysis (PCA) was conducted using the FactoMineR package, with visualization facilitated through the factoextra package. Principal components (PCs) with eigenvalues greater than 1 were considered significant, following Kaiser’s criterion. Furthermore, a hierarchical cluster analysis (HCA) was performed utilizing the hclust function (stats package) to classify stations and stages into distinct clusters. The clustering structure was visualized employing the pheatmap package to effectively illustrate relationships among measured parameters.

Satellite images and their corresponding spectral bands were downloaded from the Copernicus Open Access Hub using Sentinel‐2 data. The processing and analysis of the spectral parameters were carried out using QGIS software. This step involved the extraction and computation of vegetation and water‐related indices, to assess spatial variations in the vegetation density and water content across the study area.

## 3. Results

ANOVA (Table 1) reveals that most of the studied parameters are significantly influenced by the site, the developmental stage, as well as by their interaction in several cases. This highlights the combined effect of environmental conditions and the phenological development of the carob tree on its morphophysiological and biochemical fruit characteristics.

**TABLE 1:** Analysis of variance (ANOVA) for the effects of the geographic location and maturity stage on the carob fruit analyzed traits.

Parameter	Source	Df	Sum sq	Mean sq	*F* value	Pr (> *F*)
Length	Station	2	346.79	173.39	48.12	< 0.001
	Stage	7	658.88	94.13	26.12	< 0.001
	Station × stage	14	113.12	8.08	2.24	< 0.05

Width	Station	2	13.31	6.65	5.15	< 0.01
	Stage	7	200.50	28.64	22.18	< 0.001
	Station × stage	14	100.72	7.19	5.57	< 0.001

Thickness	Station	2	0.25	0.12	29.47	< 0.001
	Stage	7	0.68	0.10	23.00	< 0.001
	Station × stage	14	0.09	0.01	1.50	0.148

Chord	Station	2	311.54	155.77	69.22	< 0.001
	Stage	7	381.01	54.43	24.19	< 0.001
	Station × stage	14	22.34	1.60	0.71	0.754

Pod weight	Station	2	364.79	182.39	73.16	< 0.001
	Stage	7	1.07E + 03	153.49	61.56	< 0.001
	Station × stage	14	94.81	6.77	2.72	< 0.01

Seed number	Station	2	0.30	0.15	9.23	< 0.001
	Stage	7	0.22	0.03	1.98	0.078
	Station × stage	14	0.21	0.02	0.94	0.525

Aborted seeds	Station	2	0.82	0.41	2.54	0.089
	Stage	7	1.18	0.17	1.04	0.416
	Station × stage	14	2.74	0.20	1.21	0.302

Aborted seeds (%)	Station	2	12.67	6.33	4.63	< 0.05
	Stage	7	18.57	2.65	1.94	0.084
	Station × stage	14	26.66	1.90	1.39	0.194

Seed weight	Station	2	10.20	5.10	54.51	< 0.001
	Stage	7	104.23	14.89	159.18	< 0.001
	Station × stage	14	3.24	0.23	2.47	< 0.05

Seed yield	Station	2	0.79	0.39	20.80	< 0.001
	Stage	7	23.99	3.43	181.34	< 0.001
	Station × stage	14	2.98	0.21	11.25	< 0.001

L	Station	2	0.02	0.01	20.05	< 0.001
	Stage	7	0.02	0.00	5.86	< 0.001
	Station × stage	14	0.06	0.00	8.31	< 0.001

A	Station	2	173.92	86.96	14.92	< 0.001
	Stage	7	283.72	40.53	6.95	< 0.001
	Station × stage	14	391.36	27.95	4.80	< 0.001

b	Station	2	300.56	150.28	41.67	< 0.001
	Stage	7	294.19	42.03	11.65	< 0.001
	Station × stage	14	1.05E + 03	75.22	20.86	< 0.001

*C* ^∗^	Station	2	380.17	190.08	49.29	< 0.001
	Stage	7	450.28	64.33	16.68	< 0.001
	Station × stage	14	1.32E + 03	94.49	24.50	< 0.001

*H*°	Station	2	0.07	0.03	0.57	0.568
	Stage	7	0.69	0.10	1.70	0.132
	Station × stage	14	1.28	0.09	1.57	0.123

pH	Station	2	0.00	0.00	127,426.11	< 0.001
	Stage	7	0.01	0.00	171,072.86	< 0.001
	Station × stage	14	0.02	0.00	136,923.43	< 0.001

Brix	Station	2	31.63	15.81	2981.16	< 0.001
	Stage	7	32.34	4.62	870.85	< 0.001
	Station × stage	14	16.75	1.20	225.55	< 0.001

TPC	Station	2	0.28	0.14	35.76	< 0.001
	Stage	7	1.13	0.16	41.14	< 0.001
	Station × stage	14	0.26	0.02	4.73	< 0.001

TFC	Station	2	3.09	1.54	355.65	< 0.001
	Stage	7	27.04	3.86	889.68	< 0.001
	Station × stage	14	3.76	0.27	61.79	< 0.001

TCT	Station	2	9.33E + 03	4.67E + 03	2.71	0.076
	Stage	7	5.36E + 06	7.66E + 05	445.28	< 0.001
	Station × stage	14	1.01E + 06	7.20E + 04	41.91	< 0.001

TSC	Station	2	1.49E + 05	7.43E + 04	15.60	< 0.001
	Stage	7	6.28E + 06	8.97E + 05	188.26	< 0.01
	Station × stage	14	1.55E + 05	1.11E + 04	2.33	< 0.05

DPPH	Station	2	3.65E + 07	1.82E + 07	254.22	< 0.001
	Stage	7	8.75E + 07	1.25E + 07	174.20	< 0.001
	Station × stage	14	1.46E + 07	1.04E + 06	14.52	< 0.001

ABTS	Station	2	9.89E + 03	4.95E + 03	1.36	0.267
	Stage	7	1.86E + 05	2.66E + 04	7.32	< 0.001
	Station × stage	14	4.94E + 04	3.53E + 03	0.97	0.496

FRAP	Station	2	2.73	1.36	39.18	< 0.001
	Stage	7	3.56	0.51	14.64	< 0.01
	Station × stage	14	1.01	0.07	2.08	< 0.05

Regarding the morphological fruit traits, pod length and width are strongly influenced by the site, the stage, and their interaction. This indicates significant variation in these fruit dimensions depending on the geographical location and maturation stage, suggesting that differences in environmental conditions among sites directly affect fruit growth. In contrast, fruit thickness and chord show highly significant effects from both the site and stage, but the interaction is not significant, suggesting that the influence of the site and the stage on these parameters is independent.

Concerning agronomic traits, pod weight, seed weight, and seed yield were significantly influenced by all three sources of variation, indicating that productivity varied by site, stage, and their combination. Seed number was affected only by site, suggesting relative stability of this trait across stages but sensitivity to the geographic location. Aborted seeds showed no significant overall effect, except for abortion rate, which varied by site, revealing a subtle but significant geographic influence on reproductive success.

Colorimetric indices, *L* ^∗^, *a* ^∗^, *b* ^∗^, and *C* ^∗^ parameters, were significantly influenced by site, stage, and their interaction, reflecting significant variability in fruit color depending on the environment and development. In contrast, hue (*h*°) was not influenced by either the site or the stage, which demonstrates a certain stability of this colorimetric component.

Physicochemical quality attributes, represented by pH and Brix, were strongly affected by all the factors studied, reflecting marked differences between sites and stages, probably related to sugar and organic acid contents. Similarly, bioactive compounds such as TPC, TFC, TCTs, TSC, as well as antioxidant capacities measured by DPPH, ABTS, and FRAP assays, were significantly affected by the site, the stage, and their interaction. These results indicate a differentiated response of secondary metabolites depending on the environment and the developmental stage. In contrast, the ABTS assay was not influenced by either the site or the interaction, although it varied depending on the stage, suggesting an essentially developmental dynamic of this antioxidant capacity.

The high variability observed in bioactive compounds and yield parameters underscores the importance of rigorous geographical and temporal selection in order to optimize the quality and productivity of carob trees in Morocco.

### 3.1. Pomological Traits

**FIGURE 1:**
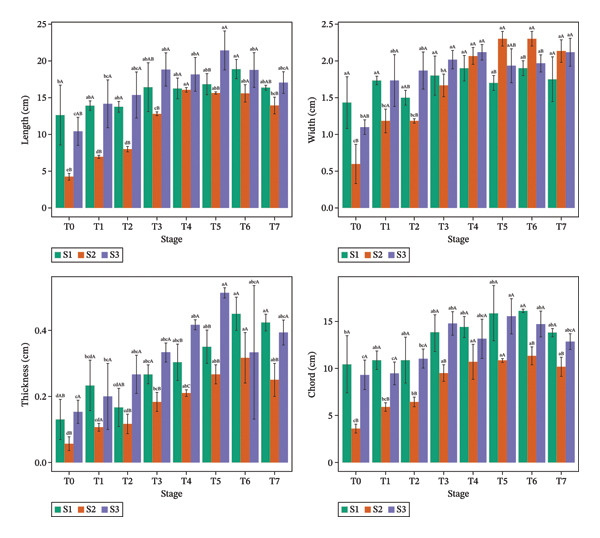
Variation of fruit morphological traits as a function of maturity stages and stations. Different lowercase letters indicate the significant differences among stages within the same station, while different uppercase letters indicate the significant differences among stations within the same stage.

Horticulture, as a scientific discipline, focuses primarily on the development of the plant material for human consumption and therapeutic use, or to meet functional and aesthetic criteria [32]. In this context, immature carob pods are products of great importance for pharmacological and dietary practices. The combined analysis of the effects of the geographical location and maturity stage (Figure 1) revealed highly significant variability in the characteristics of carob pods, as confirmed by the ANOVA results (Table 1), indicating a clear influence of environmental conditions and physiological development on the morphological quality of the fruit.

The dimensions of the pods, namely, their length, width, thickness, and chord, exhibited a consistent progression as the maturity stages advanced, attaining their maximum values at stages *T*_5_ to *T*_7_. The maximum pod length was observed at Station *S*_3_‐*T*_5_, measuring 21.43 ± 2.65 cm, while at the earlier stages (*S*_2_‐*T*_0_), it was significantly lower, measuring 4.27 ± 0.45 cm. This indicates a dynamic of sustained growth during maturity. The chord, which is an additional indicator of pod size, followed a similar trend, with maximum values recorded between *T*_5_ and *T*_7_, reaching up to 16.1 ± 0.17 cm at *S*_1_‐*T*_6_, reflecting complete maturity of the fruit.

Station *S*_3_ is distinguished by its high overall values for physical dimensions, with the exception of the width parameter, which exhibited greater significance for Station *S*_2_, particularly following Stage *T*_5_. These variations indicate that local soil and climate conditions have a favorable effect on fruit development.

**FIGURE 2:**
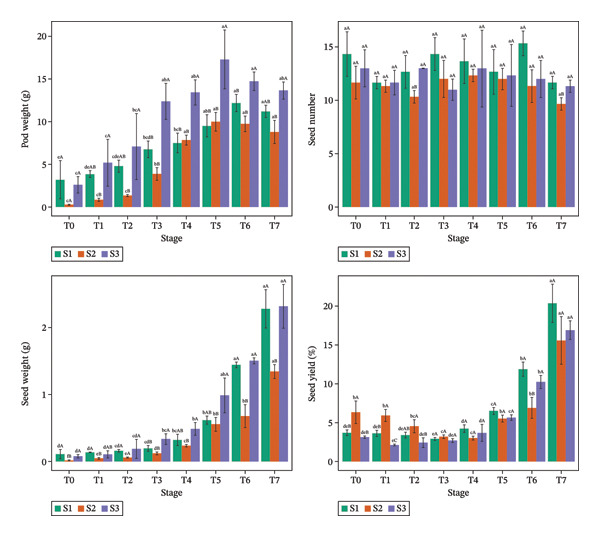
Variation of agronomical traits as a function of maturity stages and stations. Different lowercase letters indicate the significant differences among stages within the same station, while different uppercase letters indicate the significant differences among stations within the same stage.

A thorough examination of the data presented in Figure 2 reveals a gradual increase in pod weight across the three stations during the various growth stages. It is important to note that these increases follow distinct patterns. Station *S*_2_ recorded the maximum weight of 17.29 ± 3.45 g at stage *T*_5_. Furthermore, it was observed that the weight of immature pods began to decrease after growth stage *T*_5_ at most stations. This observation indicates the commencement of pod maturation, a process characterized by the water loss and the pulp lignification. However, a significant increase in seed weight was observed during the growth phases, reaching maximum values of 2.28 g, 1.34 g, and 2.31 g for Stations *S*_1_, *S*_2_, and *S*_3_, respectively.

The number of seeds per pod remained relatively stable among stations and stages, ranging from 9.67 to 15.33. However, the seed yield (%) showed a strong increase at advanced stages, reaching up to 20.34 ± 2.44% at *S*_1_‐*T*_7_, indicating an improved allocation of resources to seeds during maturity.

These results indicate that the physiological maturity of the fruit, in conjunction with local agro‐ecological conditions, exerts a pivotal role in regulating the agronomical characteristics of the unripe carob pod.

The findings of the present study are in alignment with those of previous research concerning pomological characteristics, encompassing fruit length, width, weight, and seed weight [33, 34].

### 3.2. Color Attributes

The initial maturity stage (*T*_0_) of the carob fruit exhibited higher *L* ^∗^ values, particularly at *S*_2_ (79.44 ± 6.26), indicating a lighter surface color (Table 2). Stations *S*_2_ and *S*_3_ maintained relatively high *L* ^∗^ values throughout the maturity stages. In contrast, *S*_1_ showed a marked decrease in *L* ^∗^ as maturity progressed, suggesting a darkening or reduction in the brightness of the pods as ripening progressed. This change is primarily attributed to the enzymatic oxidation of polyphenols by polyphenol oxidase (PPO) and peroxidase (POX) [13]. The differences between stations may be explained by variations in microclimatic conditions or by differences in the initial physicochemical composition of the pods.

**TABLE 2:** Effect of geographic origin and maturity stage on the color traits of the carob fruit.

Station	Stage	*L* ^∗^	*a* ^∗^	*b* ^∗^	*C* ^∗^	*h*°	pH	Brix
*S*_1_	*T*_0_	77.12 ± 2.06^aA^	−11.47 ± 1.5^abB^	18.66 ± 4.4^bA^	21.92 ± 4.53^bA^	−1.01 ± 0.05^aA^	5.72 ± 0.0^aA^	8.08 ± 0.14^bA^
*S*_2_	*T*_0_	79.44 ± 6.26^abA^	−2.4 ± 0.31^aA^	11.42 ± 3.36^cA^	11.68 ± 3.32^dB^	−1.35 ± 0.05^aB^	5.46 ± 0.0^hC^	7.5 ± 0.0^aB^
*S*_3_	*T*_0_	78.94 ± 1.91^aA^	−9.77 ± 1.33^aB^	13.78 ± 3.99^bA^	16.92 ± 4.01^bAB^	−0.94 ± 0.08^aA^	5.67 ± 0.0^dB^	6.5 ± 0.0^cC^
*S*_1_	*T*_1_	71.73 ± 1.99^bB^	−16.09 ± 0.38^cB^	25.83 ± 1.04^aA^	30.43 ± 0.95^aA^	−1.01 ± 0.02^aB^	5.72 ± 0.0^aA^	7.08 ± 0.14^dA^
*S*_2_	*T*_1_	83.42 ± 0.56^aA^	−5.29 ± 0.05^abA^	4.81 ± 0.69^dB^	7.16 ± 0.51^eB^	−0.74 ± 0.06^aA^	5.53 ± 0.0^eC^	4.5 ± 0.0^fC^
*S*_#_	*T*_1_	73.58 ± 0.81^cB^	−15.65 ± 0.3^bB^	24.46 ± 0.55^aA^	29.04 ± 0.62^aA^	−1.0 ± 0.0^abB^	5.67 ± 0.0^cB^	6.0 ± 0.0^eB^
*S*_1_	*T*_2_	74.1 ± 0.66^abA^	−15.2 ± 0.2^cB^	26.2 ± 0.82^aA^	30.29 ± 0.8^aA^	−1.04 ± 0.01^aB^	5.62 ± 0.0^dC^	8.5 ± 0.0^aA^
*S*_2_	*T*_2_	76.68 ± 0.21^bA^	−14.56 ± 0.09^bB^	23.72 ± 0.33^aA^	27.83 ± 0.32^aA^	−1.02 ± 0.0^aAB^	5.63 ± 0.0^bB^	6.5 ± 0.0^bB^
*S*_3_	*T*_2_	77.23 ± 2.22^abcA^	−11.23 ± 1.88^aA^	16.04 ± 4.38^bB^	19.6 ± 4.67^bB^	−0.95 ± 0.05^aA^	5.83 ± 0.0^bA^	6.0 ± 0.0^eC^
*S*_1_	*T*_3_	75.07 ± 0.75^abA^	−11.07 ± 0.26^abA^	17.1 ± 0.72^bB^	20.37 ± 0.75^bB^	−1.0 ± 0.01^aAB^	5.64 ± 0.0^cA^	8.0 ± 0.0^bA^
*S*_2_	*T*_3_	76.75 ± 0.99^bA^	−14.57 ± 0.24^bB^	24.07 ± 0.28^aA^	28.13 ± 0.34^aA^	−1.03 ± 0.0^aB^	5.57 ± 0.0^dC^	6.5 ± 0.0^bC^
*S*_3_	*T*_3_	77.65 ± 1.36^abA^	−10.03 ± 1.0^aA^	14.57 ± 2.71^bB^	17.7 ± 2.81^bB^	−0.96 ± 0.04^aA^	5.58 ± 0.0^eB^	7.5 ± 0.0^bB^
*S*_1_	*T*_4_	73.39 ± 2.52^abB^	−10.67 ± 0.61^aA^	17.42 ± 1.84^bA^	20.43 ± 1.89^bA^	−1.02 ± 0.02^aA^	5.56 ± 0.0^fB^	7.5 ± 0.0^cA^
*S*_2_	*T*_4_	73.72 ± 0.28^bcB^	−3.15 ± 11.21^aA^	15.61 ± 0.07^bA^	18.37 ± 0.08^bcA^	−0.34 ± 1.17^aA^	5.58 ± 0.0^cA^	6.0 ± 0.0^cC^
*S*_3_	*T*_4_	78.29 ± 0.69^abA^	−9.83 ± 0.44^aA^	14.66 ± 1.16^bA^	17.65 ± 1.21^bA^	−0.98 ± 0.02^aA^	5.56 ± 0.0^fC^	6.25 ± 0.0^dB^
*S*_1_	*T*_5_	74.47 ± 0.56^abB^	−11.24 ± 0.17^abB^	18.26 ± 0.42^bA^	21.44 ± 0.44^bA^	−1.02 ± 0.0^aB^	5.36 ± 0.0^gC^	6.0 ± 0.0^eA^
*S*_2_	*T*_5_	69.84 ± 1.05^cC^	−11.64 ± 0.08^abB^	18.26 ± 0,11^bA^	21.65 ± 0.12^bA^	−1.0 ± 0.0^aAB^	5.53 ± 0.0^fA^	5.17 ± 0.29^eB^
*S*_3_	*T*_5_	78.94 ± 1.79^aA^	−9.63 ± 0.65^aA^	14.76 ± 1.44^bB^	17.62 ± 1.56^bB^	−0.99 ± 0.01^aA^	5.53 ± 0.0^hB^	5.0 ± 0.0^fB^
*S*_1_	*T*_6_	72.48 ± 0.76^bB^	−10.17 ± 0.26^aB^	21.28 ± 0.53^abA^	23.58 ± 0.59^bA^	−1.12 ± 0.0^bA^	5.7 ± 0.0^bB^	8.0 ± 0.0^bA^
*S*_2_	*T*_6_	74.81 ± 0.92^bcA^	−8.03 ± 0.36^abA^	15.21 ± 0.62^bC^	17.2 ± 0.65^cC^	−1.09 ± 0.02^aA^	5,52 ± 0.0^gC^	5.5 ± 0.0^dC^
*S*_3_	*T*_6_	74.73 ± 0.48^bcA^	−8.75 ± 0.31^aA^	17.4 ± 1.05^bB^	19.49 ± 0.81^bB^	−1.1 ± 0.04^bA^	5.9 ± 0.0^aA^	8.0 ± 0.0^aB^
*S*_1_	*T*_7_	74.32 ± 0.17^abB^	−12.62 ± 0.24^bB^	20.58 ± 0.83^bA^	24.14 ± 0.83^bA^	−1.02 ± 0.01^aA^	5.6 ± 0.0^eB^	7.53 ± 0.05^cA^
*S*_2_	*T*_7_	76.64 ± 1.12^bA^	−8.6 ± 1.91^abA^	16.32 ± 0.58^bB^	18.48 ± 1.36^bcB^	−1.09 ± 0.08^aA^	5.82 ± 0.0^aA^	6.4 ± 0.0^bC^
*S*_3_	*T*_7_	77.44 ± 0.38^abA^	−11.02 ± 0.33^aAB^	16.38 ± 0.68^bB^	19.74 ± 0.74^bB^	−0.98 ± 0.01^aA^	5.55 ± 0.0^gC^	6.5 ± 0.0^cB^

*Note:* Different lowercase letters indicate the significant differences among stages within the same station, while different uppercase letters indicate significant differences among stations within the same stage.

The *a* ^∗^ value represents the red–green component of color in the CIELAB system, with positive values corresponding to red color and negative values to green color. In our study, *a* ^∗^ values were notably negative during early maturation in Stations *S*_1_ and *S*_2_. The fruits cultivated in Station *S*_2_ showed a greater decrease in *a* ^∗^ values from stage *T*_0_ to *T*_3_ compared to *S*_1_ and *S*_3_, possibly due to faster fruit development in *S*_2_, which could be influenced by local microclimatic conditions during this period. At later maturity stages, the fruits retained negative *a* ^∗^ values compared to the initial stage, indicating an intensification of green coloration before the transition to brownish tones. This evolution is likely related to chlorophyll degradation and the subsequent accumulation of brown pigments [35]. At the final maturity stage (*T*_7_), both *a* ^∗^ and *b* ^∗^ values were higher in *S*_1_ compared to the other stations, while the *L* ^∗^ value was lower. This combination may indicate a more advanced ripening stage, potentially influenced by soil composition or other local environmental factors. The *b* ^∗^ values increased markedly at advanced maturity stages, reflecting the development of yellow–brown tones characteristic of ripe carob pods. This pattern was further supported by the chroma (*C* ^∗^), which reached its highest values at peak maturity (*T*_7_), indicating higher color saturation. The hue angle (*H*°) remained relatively stable, with slightly negative values around −1, suggesting a predominance of brownish‐green tones typical of mature carob.

The pH of carob pods in this study remained within a relatively narrow range (5.36–5.83) across all maturity stages and geographic origins, reflecting the low acidity character of the fruits. At the initial maturity stage (*T*_0_), pH values showed clear differences between stations, Stations *S*_1_ and *S*_3_ recorded the highest initial pH (5.72 and 5.67, respectively), whereas *S*_2_ had the lowest value (5.46). During the intermediate stages (*T*_1_–*T*_4_), pH values remained relatively stable in *S*_1_ and *S*_3_, while *S*_2_ showed a slight increase after *T*_2_, indicating a gradual loss of acidity. This may suggest that environmental conditions in *S*_2_ promote a faster reduction in organic acids during this ripening period, possibly due to increased respiration rates and acid metabolism [36]. By the final stages (*T*_5_–*T*_7_), pH values increased in *S*_2_ and *S*_3_, likely due to the breakdown of malic and citric acids during ripening [37, 38]. These results are in agreement with Benchikh et al. [39], who reported that carob fruits exhibit a decrease in titratable acidity as the fruit matures, leading to an increase in pH.

The °Brix values increased with maturity in all stations, showing that carob pods accumulate more sugars as they ripen. This rise is mainly due to the breakdown of complex carbohydrates into simple sugars and the concentration effect resulting from water loss [40]. *S*_1_ generally had higher °Brix values than *S*_2_ and *S*_3_, suggesting better conditions for sugar formation, possibly related to environmental conditions. These results show that both the maturity stage and growing location influence the sweetness of carob fruits. At the initial maturity stage (*T*_0_), carob pods from *S*_1_ showed the highest TSS value (8.08°Brix) wish may be due to the advanced physiological development even at the start of the sampling period. In contrast, *S*_3_ had the lowest value (6.50°Brix), suggesting that sugar accumulation was still at an early stage. These differences at *T*_0_ may be linked to climatic factors such as temperature and light availability, which affect photosynthesis and the movement of sugars within the plant. During the intermediate stages (*T*_1_–*T*_4_), TSS in *S*_1_ remained relatively high (7.08–8.50°Brix). In contrast, *S*_2_ showed a marked decrease, reaching the lowest value recorded in the study at *T*_1_ (4.51°Brix), which may indicate slower sugar accumulation or possible dilution due to the higher moisture content. Station *S*_3_ maintained moderate levels (6.00–6.50°Brix) throughout this period. The lower values observed in *S*_2_ may reflect environmental conditions that limit sugar production or its redistribution within the fruit. By the final maturity stages (*T*_5_–*T*_7_), TSS increased in all stations, reflecting the natural accumulation of sugars as the pods ripened. Station *S*_1_ again recorded the highest value at *T*_2_ (8.55°Brix), showing a strong ability to produce and retain sugars. On the other hand, *S*_3_ and *S*_2_ ended with slightly lower values (8.00 and 7.53°Brix), respectively, which could be related to differences in the pod dry matter content or genetic factors influencing sugar metabolism.

These differences highlight the importance of choosing both the right harvest time and the appropriate production area to achieve optimal sweetness and quality for processing, especially in the case where the sugar content is key to market value and consumer appeal.

### 3.3. Biochemical Traits

**FIGURE 3:**
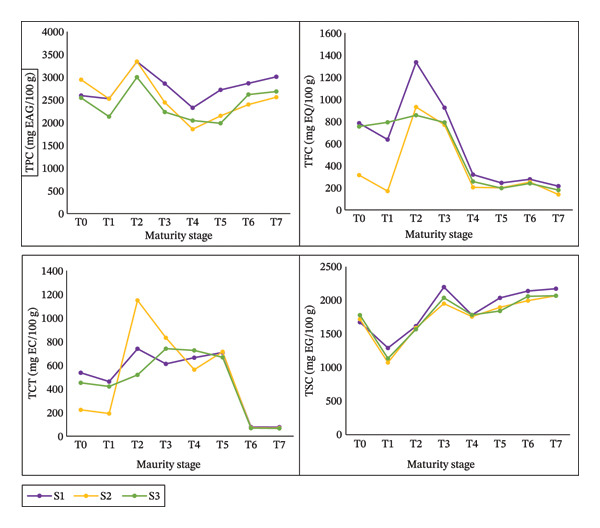
Effect of geographic origin and maturity stage on the biochemical traits of the carob fruit.

The results revealed that the carob biochemical traits varied significantly across maturity stages and geographical origins (Figure 3). Regarding the TPC, the results revealed that this parameter fluctuated across fruit maturity stages and environmental conditions. Several studies reported that such fluctuation in the fruit phenolic concentrations was consistent with the physiological roles of phenolic compounds in fruit, which act as protective compounds against oxidative stress in plant tissues, UV radiation, and pathogens during the fruit development stages [41]. Generally, the early stages show high phenolic syntheses due to metabolic activity in young tissues, followed by dilution during rapid fruit development and occasional concentration effects due to water loss at later maturity stages [42].

For TPC, the highest concentration was detected at the intermediate maturity stage (*T*_2_) in all experimental stations, with values reaching 3339 ± 456 mg EAG/100 g in *S*_1_ and 3342 ± 377 mg EAG/100 g in *S*_2_. Furthermore, significant interstation variations were detected at specific maturity stages, underscoring the influence of geographic origin. Notably, at most stages, the highest TPC values were detected at experimental Station *S*_1_. The results presented in Figure 3 also revealed a highly significant effect of environmental conditions on carob fruit during maturation. The highest levels of the TPC were detected at the *S*_2_ station, whereas Station *S*_3_ revealed the lowest values. In agreement with these results, Rivero et al. [43] reported the high significant effect of the environmental factors such as soil composition, temperature fluctuations, and precipitation patterns on the plant biochemical properties and revealed that the drier area can generate abiotic stress responses in plants, leading to the elevated synthesis of secondary metabolites such as phenolic compounds. Similarly, Ayaz et al. [44] demonstrated that the accumulation of carob phenolic compounds is influenced by the climatic conditions, with fruit originating from areas characterized by elevated temperatures exhibiting the highest TPC levels. In parallel, Loullis and Pinakoulaki [45] reported that sunlight exposure during pod maturation enhances the phenolic content in carob fruits. This observation is consistent with the fact that phenolic compounds, as secondary metabolites with protective functions, are highly responsive to environmental cues and stress signals, including light exposure [46]. Accordingly, both preharvest and postharvest factors can modulate phenolic accumulation, leading to either enrichment or degradation of these compounds in fruits and vegetables. In particular, preharvest conditions such as temperature, soil characteristics, harvest timing, and especially light intensity can significantly influence phenolic biosynthesis. Postharvest handling practices, including processing operations and storage conditions, as well as shelf‐life environments, further contribute to the regulation and stability of phenolic compounds [47, 48]. These environmental influences may therefore explain the sustained high phenolic levels observed in certain experimental stations in the present study, even at advanced stages of carob pod development.

Regarding the carob TFC, the data showed a distinct stage‐dependent trend, with maximum levels at early (*T*_0_ stage) to mid‐maturity (*T*_2_ stage) in *S*_1_ and *S*_3_, with values reaching 1335 ± 187 mg EQ/100 g at *T*_2_ in *S*_1_, while *S*_2_ consistently exhibited lower values at these stages. The disparity between stations was particularly pronounced at *T*_0_, where *S*_1_ and *S*_3_ exceeded *S*_2_ by more than twofold. Moreover, the lowest TFC values were recorded during the final stages (*T*_5_–*T*_7_), all below 250 mg EQ/100 g, with the highest values observed in Station *S*_1_.

For TCTs, the results revealed that the *S*_3_ station tended to show lower tannin levels across most stages, with notable interstation differences at *T*_0_ and *T*_3_. A sharp peak was observed at *T*_2_ in *S*_2_ (1149 ± 55 mg EC/100 g), significantly higher than both *S*_1_ and *S*_3_ at the same stage. Conversely, the lowest TCT values were observed at the final stages, *T*_6_ and *T*_7_, with an average of 70 mg EC/100 g.

In contrast, the data reported that the TSC in carob fruit increased during the late maturity stages, with the highest values generally observed at *T*_7_. However, unlike phenolic compounds, the variation in sugar accumulation showed limited interstation variability at advanced maturity.

Analysis of TFC and TCTs in carob fruit revealed pronounced variations throughout the maturity process, with distinct patterns between the studied experimental sites. Similar trends have been reported by Avallone et al. [49] who noted that carob pods generally exhibit a peak in flavonoid and tannins concentrations at intermediate maturity stages followed by a progressive reduction. Comparable observations in grapes and other polyphenol‐rich fruits have been reported by Downey et al. [50] where flavonoid accumulation during maturation shows sensitivity to environmental conditions, supporting the hypotheses that TFC and TSC are both stage‐ and climate‐dependent. Similarly, Loullis and Pinakoulaki [45] reported that the carob tannin content in the fruit’s peaks before full maturity, particularly under hot, dry environmental conditions, which matches *S*_2_’s data. Similar patterns are found in other legumes, where condensed tannins decrease with pod ripening as lignification progresses [51]. The data revealed that at the *S*_1_ station, the maximum TFC values were reported, particularly at *T*_2_ and *T*_3_. This variation can be due to the differences in solar exposure and microclimate. In this sense, Fadel et al. [52] revealed that the warmer and sunnier sites often induce higher flavonoid biosynthesis. In the same way, *S*_2_’s extremely high TCT production at *T*_2_ stage may be related to environmental stress conditions that stimulate tannin biosynthesis as part of the plant’s defense strategy [53].

### 3.4. Antioxidant Activity

**FIGURE 4:**
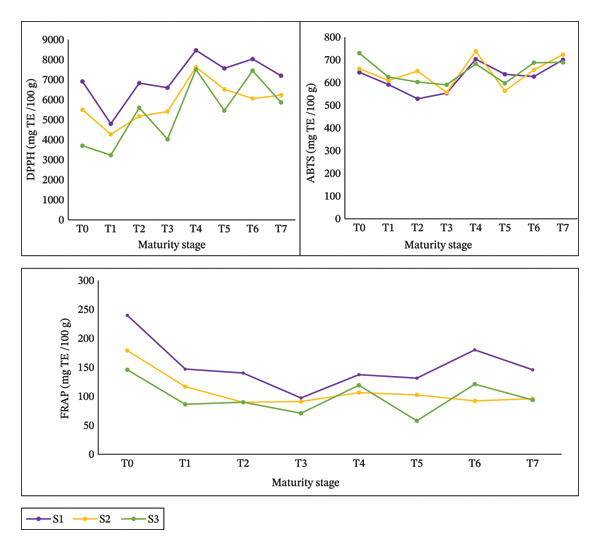
Effect of geographic origin and maturity stage on the antioxidant activity of the carob fruit.

The antioxidant activities of the carob fruit measured via DPPH, ABTS, and FRAP assays revealed marked variations attributable to both the maturity stage and geographic origin (Figure 4). The highest values of the DPPH radical‐scavenging activity were generally recorded at *T*_4_ across stations, with a peak of 8469 ± 201 mg TE/100 g in the *S*_1_ station. In contrast, earlier fruit maturity stages, such as *T*_0_ and *T*_1_, displayed markedly lower DPPH activity. Interstation differences were evident at several stages, with the highest values detected in the *S*_1_ stations. On the other hand, ABTS radical cation decolorization activity presented a more stable profile across experimental stations, indicating the dominance of maturity stage over geographic origin in determining ABTS activity. Notably, the highest ABTS values were detected at *T*_4_ and *T*_7_ stages in all stations, with the values reaching 700 mg TE/100 g. The relative uniformity among stations suggests that the compounds responsible for ABTS scavenging, potentially including both hydrophilic and lipophilic antioxidants, are less sensitive to station environmental variability than those affecting DPPH activity.

Regarding the FRAP of carob fruit during maturity stages, the results exhibited a high variability at early maturity stages, with strong interstation differences at *T*_0_ (239 ± 50 mg TE/100 g at *S*_1_ and 145 ± 10 mg TE/100 g at *S*_3_). Such differences diminished at later stages, with most stations converging to similar FRAP values during the *T*_4_ and *T*_7_ stages, indicating that the developmentally driven accumulation of reducing agents overshadowed site‐specific influences at advanced maturity. Interestingly, in the late stage (*T*_6_), data revealed that the *S*_1_ station recorded a higher FRAP activity (180 ± 5 mg TE/100 g) compared to *S*_2_ and *S*_3_, hinting at a persistent geographical effect under specific environmental contexts.

The same variation during the ripening stages was detected in the antioxidant activity. In all experimental stations, DPPH activity was generally highest at the final fruit developmental stages but also showed considerable early stage values, although the magnitude and pattern of DPPH change varied among sites. In agreement with these findings, Kyriacou et al. [13] reported a clear decline in DPPH radical‐scavenging capacity as carob fruit ripens, with the sharpest drop between development stages, and revealed that the carob fruit from higher elevation sites showed higher DPPH activity, indicating an environmental influence. Regarding the ABTS and FRAP antioxidant activity, Benchikh et al. [54] found that the unripe carob pods are richer in phenolic compounds and exhibit stronger ABTS and FRAP antioxidant activity, with reported IC_50_ values like 54.92 ± 0.23 μg/mL for ABTS, further demonstrating the decline in antioxidant potency upon fruit maturity. The high significant variation in the antioxidant activity of carob fruit during maturation across different geographic conditions align with the finding of Laaraj et al. [5], who reported that the antioxidant activity occurred during the early ripening stage, with significant variation observed between the geographic locations. They attributed the site‐dependent differences to the geographic modulation of the decline in phenolic compounds, which accompany fruit growth and maturation.

### 3.5. Multivariate Analyses

PCA carried out on all measured physical and biochemical parameters revealed significant structuring of the data according to the geographical origin (*S*_1_, *S*_2_, *S*_3_) and maturity stage (*T*_0_ to *T*_7_) of the carob fruits (Table 3). The first six PCs together explain 83.64% of the total variance, with the first three axes contributing 27.99%, 19.63%, and 13.56%, respectively, reflecting PCA’s ability to summarize the initial information.

**TABLE 3:** Eigenvectors of principal components from PCA analysis based on the variation of the analyzed traits under different geographic locations and maturity stages.

Parameter	PC1	PC2	PC3	PC4	PC5	PC6
Length	0.875	0.296	−0.256	−0.159	0.128	−0.016
Width	0.784	0.010	−0.383	0.066	−0.055	0.173
Thickness	0.913	0.146	−0.187	−0.007	0.075	−0.156
Chord	0.846	0.333	−0.030	−0.213	0.155	−0.119
Pod weight	0.931	0.003	−0.190	0.031	0.078	−0.151
Seed number	0.123	0.340	0.313	−0.573	0.528	0.066
Aborted seeds	−0.148	0.287	−0.325	0.218	0.765	−0.033
Aborted seeds (%)	−0.236	0.179	−0.494	0.653	0.270	0.165
Seed weight	0.952	0.066	0.013	0.246	−0.058	0.001
Seed yield	0.544	−0.293	0.469	0.515	−0.226	−0.089
L	−0.366	−0.607	−0.077	0.090	0.319	−0.387
a	0.083	−0.860	0.192	−0.194	0.211	0.068
b	−0.019	0.894	0.038	0.119	−0.342	0.163
*C* ^∗^	−0.041	0.904	−0.042	0.107	−0.327	0.170
H	0.022	−0.249	−0.449	−0.353	0.074	0.547
pH	0.005	0.313	0.063	0.495	0.431	0.452
Brix	0.018	0.530	0.673	−0.030	0.188	−0.011
TPC	−0.316	0.325	0.571	0.402	0.120	−0.312
TFC	−0.597	0.680	−0.081	−0.108	0.222	−0.112
TCT	−0.324	0.419	−0.415	−0.470	−0.071	−0.190
TSC	0.694	0.078	0.262	0.097	0.169	−0.296
DPPH	0.590	0.119	0.488	−0.318	0.076	0.168
ABTS	0.238	−0.489	0.261	0.183	0.073	0.480
FRAP	−0.184	0.066	0.848	−0.196	0.061	0.278
Eigenvalue	6.716	4.712	3.255	2.222	1.754	1.415
Variance (%)	27.985	19.634	13.562	9.257	7.310	5.895
Cumulative variance (%)	27.985	47.619	61.181	70.438	77.748	83.643

The first component (PC1), which explained 27.99% of the variability, was mainly linked to pod and seed morphological parameters, notably seed and pod weight, pod thickness, length, and width, as well as seed yield. It was also positively correlated with total soluble sugar content (TSC) and antioxidant activity measured by DPPH and negatively correlated with TFC and certain colorimetric parameters (L). Therefore, this component reflects a gradient of physical and biochemical fruit development and is associated with greater biomass valorization and sugar accumulation [55]. The second component (PC2), which explained 19.63% of the variance, was dominated by color parameters (b, *c* ^∗^, L, and a values), flavonoids (TFC), and condensed tannin (TCT) contents. This component seems to reflect pigment variations associated with fruit ripening and thus differentiates samples according to their level of phenolic development and visual appearance [56]. The third component (PC3), representing 13.56% of the variance, was strongly positively correlated with antioxidant activity (FRAP), TPC, sugar content (Brix), and DPPH. It can be used to distinguish fruits according to their richness in bioactive antioxidant compounds and could reflect an advanced stage of ripening characterized by a high accumulation of secondary metabolites [13].

Thus, this PCA revealed significant variability in the quality of carob fruits, depending on their origin and stage of development. The first component highlighted revealed morphological characteristics, sugar content, and phenolic compounds as the main discriminating factors across samples. These findings suggest that local environmental conditions and the physiological stage of carob fruits significantly influenced the nutritional and functional attributes [57].

**FIGURE 5:**
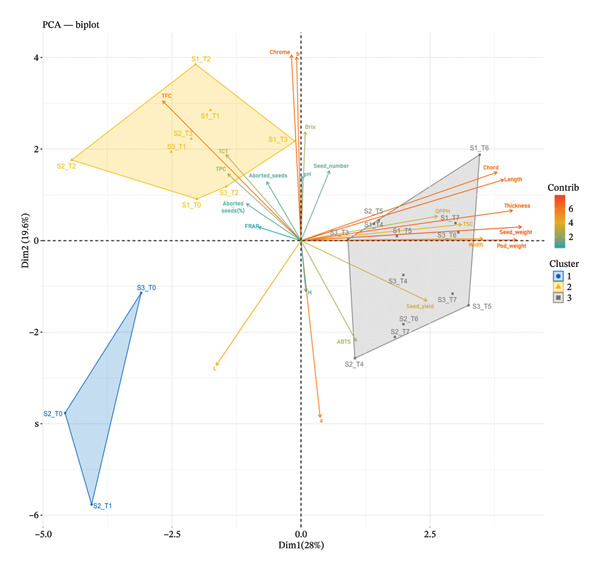
Principal component analysis (PCA) biplot of carob fruit colorimetric and biochemical traits from different sites and maturity stages.

The graphical presentation of PCA was carried out on the carob fruit data, confirming the results reported in Table 3 and revealing a clear structuring of the samples according to geographical origin (*S*_1_, *S*_2_, *S*_3_) and maturity stage (*T*_0_ to *T*_7_) (Figure 5). The first two PCs (PC1 and PC2) explained 47.62% of the total variance, reflecting the main axes of variation in the physicochemical and biochemical characteristics studied.

The first component (PC1), which explained 27.98% of the variance, was strongly correlated with morphological parameters such as length, width, thickness, pod weight, seed weight, and seed yield. This represents a gradient of physical fruit development. Samples located on the positive side of this axis (notably those from site *S*_3_ at advanced stages such as *T*_6_) were distinguished by larger, heavier pods, reflecting advanced maturity. Conversely, individuals located on the negative side (such as *S*_2__*T*_0_ and *S*_2__*T*_1_) corresponded to the early stages, characterized by low biomass. The second component (PC2), which explained 19.63% of the variance, was mainly linked to the biochemical and functional properties of the fruit, in particular, TFC, TPC, antioxidant activity (FRAP and DPPH), and certain color parameters (*C* ^∗^, and *b* ^∗^ value). Samples belonging to intermediate stages (such as *S*_1__*T*_2_ and *S*_2__*T*_2_) were strongly correlated with this axis, indicating a greater antioxidant compound richness and color intensity. In contrast, more advanced stages of ripening (such as *S*_3__*T*_6_) are more closely associated with increased fruit size and weight, but less so with biochemical richness.

Thus, the PCA graph highlights a clear differentiation of samples according to their stage of development and geographical origin, showing that young fruits are biochemically poor and morphologically underdeveloped, whereas mature fruits display significant biomass, with biochemical composition varying according to the site. Site *S*_3_ seemed to favor greater morphological development, whereas sites *S*_1_ and *S*_2_ showed enhanced accumulation of bioactive compounds at intermediate stages of maturity.

**FIGURE 6:**
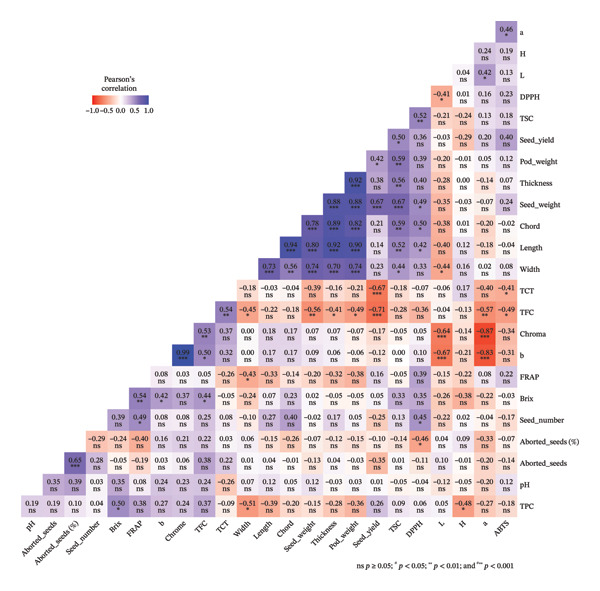
Pearson’s correlation heatmap among morphological, colorimetric, and biochemical traits of carob fruits from different sites and maturity stages.

To understand the relationship between all changes during maturation in the carob fruit under Moroccan environmental conditions, a correlation test was performed using the Pearson model (Figure 6). The results revealed significant relationships among all measured traits, including morphological, biochemical, and antioxidant properties, highlighting the interdependence of physical development and metabolic activity during the maturation of carob fruits. Significant positive correlations were revealed between the morphological traits seed weight, pod weight, pod length, width, and thickness, with a correlation coefficient ranging between *r* = 0.73 (*p* < 0.001) and *r* = 0.94 (*p* < 0.001), indicating that carob fruit physical properties varied proportionally throughout maturation. This finding reflects a coordinated growth mechanism between the pod and seed compartments of the carob fruit. Similarly, Ref. [58] revealed that the changes in the carob morphological traits likely result from the simultaneous accumulation of reserve substances, physical cell enlargement, and pod lignification occurring during the transition from immature to mature stages. Among the carob fruit colorimetric parameters, the data reported that the fruit redness (*a* ^∗^) and chromaticity (*C* ^∗^) were positively correlated with the fruit TFC and TCTs, with the coefficient *r* = 0.54–0.73 (*p* < 0.01, confirm that pigment development in the carob fruit is related to the accumulation of phenolic compounds. These results suggest that color changes during maturation can act as reliable visual indicators of the phenolic content, as reported in various fruit trees, including carob [59]. However, the fruit lightness (*L* ^∗^) and hue angle (*h*°) were inversely correlated with these compounds, reflecting the typical darkening of carob pods associated with oxidative polymerization of phenolics during ripening.

Regarding the carob biochemical, the correlation test reported a significant positive relationship between the TSC and the fruit morphological traits, including fruit dimensions (*r* > 0.5), chord (*r* = 0.59), seed weight (*r* = 0.67), thickness (*r* = 0.56), pod weight (*r* = 0.59), and seed yield (*r* = 0.56). This indicates that reduced larger pods with more biomass invest more in sugar accumulation. In agreement with these results, several studies on various carob cultivars and under different environmental conditions revealed the increase in the carob fruit sugar content during fruit ripening [60]. Furthermore, the correlation data reported a significant positive relationship between fruit maturation effects on the flavonoid content and fruit colorimetric properties such as chroma and *b* ^∗^, with a correlation coefficient higher than 0.5. In the same way, the variation in the carob seed yield during fruit maturation was negatively correlated with total flavonoids (*r* = −0.71; *p* < 0.001) and the total tannin content (*r* = −0.67; *p* < 0.001). This indicates the relationship aligns with the studies of Zhang et al. [61] who revealed the metabolic shift from active secondary metabolite synthesis at early developmental stages to carbohydrate accumulation and structural growth at advanced stages. These results support the hypothesis that immature carob pods represent the most suitable stage for obtaining high levels of bioactive compounds with potential nutraceutical value [1].

### 3.6. Remote Sensing–Based Analysis of Vegetation Density and Water Content

#### 3.6.1. Evaluation of Vegetation of Carob Tree Sampling Areas Based on NDVI Analysis

**FIGURE 7:**
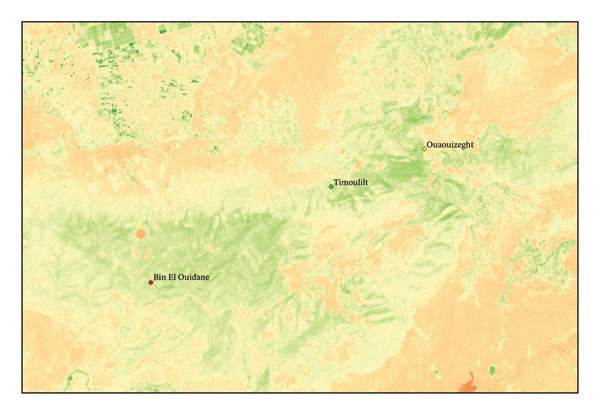
Spatial distribution of vegetation density levels in the study area based on the NDVI map.

The NDVI, derived from the red (B4) and NIR (B8) bands of Sentinel‐2 imagery, was used to characterize the vegetation cover in the three sampling areas: Timoulilt, Bin El Ouidane, and Ouaouizeght (Figure 7). The rationale for using NDVI was to provide an objective geospatial description of the vegetation context of the sampling sites, rather than establishing a direct statistical relationship with phytochemical parameters. The mean NDVI values obtained were 0.353, 0.308, and 0.270, respectively.

All three values are positive and fall within the range of 0.27–0.35, indicating active vegetation cover and relative canopy greenness in the studied areas, corresponding to the green zones on the NDVI map. According to the NDVI color classification, green areas represent the dense vegetation, yellow areas correspond to the moderate vegetation, and red areas indicate the bare soil or nonvegetated surfaces (Figure 7). Therefore, the three sampling sites are located in areas of dense vegetation, reflecting vigorous and healthy vegetation dominated by carob trees.

The highest NDVI value recorded in Timoulilt (0.35) suggests particularly vigorous vegetation, likely influenced by favorable edaphic and microclimatic conditions such as soil moisture, altitude, and exposure. These local conditions may support vegetative growth and provide a useful ecological context for interpreting the characteristics of the collected fruits. In Bin El Ouidane (0.31), the intermediate NDVI value indicates balanced vegetation cover, reflecting relatively stable environmental conditions and a moderate‐to‐good fruit quality. Conversely, the slightly lower NDVI observed in Ouaouizeght (0.27) may point to relatively lower canopy greenness or moderate environmental constraints, possibly related to soil characteristics or climatic conditions.

Although NDVI was not statistically associated with phytochemical parameters in the present study, its inclusion remains relevant because it documents the environmental heterogeneity of the sampling zones. Therefore, NDVI should be considered a complementary environmental descriptor, not a direct predictor of sugar content, phenolic compounds, or other biochemical traits.

Overall, the spatial NDVI analysis confirms that all three sampling regions fall within areas of active and dense vegetation, which supports the good vitality of the carob trees and the potentially high quality of the collected fruits. This geospatial approach therefore provides valuable contextual information for describing the collection areas and for supporting the ecological interpretation of carob fruit variability, without implying a causal relationship with phytochemical composition.

#### 3.6.2. Evaluation of Water Availability of Vegetation in Carob‐Producing Sampling Areas Using Spectral Indices

To assess the water status and physiological vigor of vegetation in the study sites (Timoulilt, Ouaouizeght, and Bin El Ouidane), two spectral indices used are the NDWI and the NDWI Geo. These indices provide information on the water content of vegetation and soil, which is particularly relevant for the carob tree, a xerophytic Mediterranean species growing under semiarid conditions. The objective of including these indices was to characterize the water‐related environmental context of the sampling areas and to complement the description provided by the NDVI.

**FIGURE 8:**
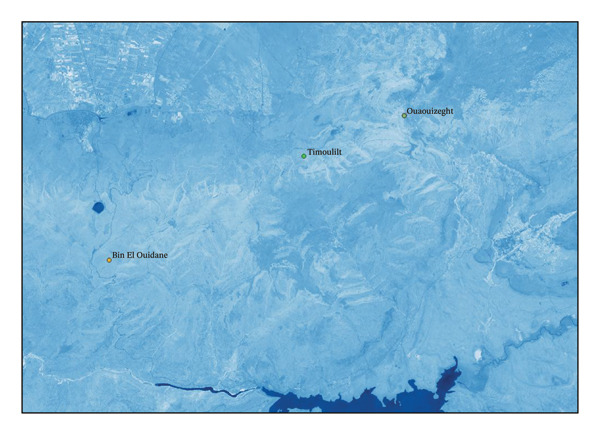
Spatial distribution of the water content in the study area based on the NDWI map.

The NDWI is an index that estimates the water content of vegetation and soil using visible and NIR spectral bands. Positive NDWI values indicate the high vegetation water content, while negative values reflect water stress or low hydration of the vegetation. In this study, the NDWI values were negative in all three sites; Timoulilt with −0.334, Bin El Ouidane with −0.314, and Ouaouizeght with −0.297, confirming a generally limited water status, typical of semiarid environments (Figure 8). Timoulilt, which recorded the lowest NDWI value, showed the most pronounced relative water limitation among the three sites. In contrast, Ouaouizeght, with the least negative NDWI value, showed comparatively better water‐related conditions, while Bin El Ouidane exhibited intermediate conditions. These differences are useful for describing the environmental variability among the sampling areas.

**FIGURE 9:**
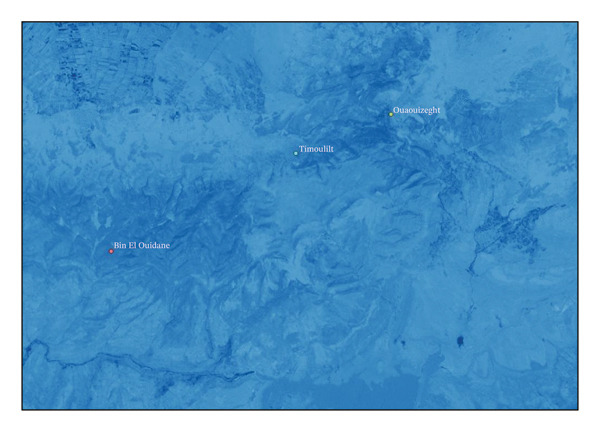
Spatial distribution of the water content in the study area based on the NDWI Geo map.

The NDWI Geo showed a positive value but was lowest with 0.0606, 0.0609, and 0.0564 for Timoulilt, Ouaouizeght, and Bin El Ouidane, respectively (Figure 9). Ouaouizeght site exhibited the highest value, suggesting slightly more favorable water conditions that support vegetative growth and the development of higher‐quality pods. Timoulilt and Bin El Ouidane showed slightly lower values, indicating more limited water availability, which could affect canopy density and pod morphology.

Although NDWI and NDWI Geo were not statistically associated with phytochemical parameters, their inclusion remains justified because they provide complementary geospatial information on water‐related environmental conditions. These indices are not presented as direct explanatory variables for fruit biochemical composition, but as descriptive indicators of the sampling environment.

The combined use of these two indices allows for the characterization of regional differences in water availability. This information strengthens the environmental description of the carob‐producing areas and helps contextualize the sampling conditions, while avoiding any unsupported causal interpretation regarding fruit phytochemical quality.

## 4. Conclusions

The present study offers comprehensive insights into the effects of geographic origin and fruit maturity stage on the morphological, biochemical, and bioactive properties of immature carob (*C. siliqua* L.) fruits growing in central Morocco’s environmental conditions. The results highlighted that both factors, along with their interaction, had a significant effect on the majority of the analyzed parameters. Globally, the fruit morphological traits increased progressively throughout fruit development, whereas the accumulation of bioactive compounds such as total polyphenols, flavonoids, and tannins peaked at the early to intermediate stage (*T*_2_) before declining as the fruits matured. According to the multivariate analysis, distinct clustering patterns according to geographical locations and the maturity stages confirm the strong dependence of carob fruit quality on environmental conditions and the development phase. These findings underscore the importance of selecting appropriate harvest times and suitable growing regions to improve the nutritional value and functional potential of carob fruits.

## Author Contributions

Salah Laaraj: conceptualization, methodology, data curation, writing–original draft preparation, and writing–review and editing. Chaimae El‐Rhouttais: conceptualization, methodology, software, investigation, resources, and writing–original draft preparation. Hicham Ouhakki: investigation, software, and writing–original draft preparation. Aziz Tikent: formal analysis, data curation, and writing–review and editing. Sofia Zazouli: software and formal analysis. Atman Adiba: conceptualization, methodology, validation, and writing–original draft preparation. Mohamed Kouighat: investigation, resources, and writing–original draft preparation. Abdellatif Boutagayout: methodology, formal analysis, and data curation. Anas Hamdani: validation, formal analysis, and writing–original draft preparation. Mohamed Lamsiah: software, formal analysis, and investigation. Ayour Jamal: software, writing–review and editing. Mohammed Mitache: formal analysis, writing–review and editing, and visualization. Souad Salmaoui: validation, writing–review and editing, visualization, supervision, and project administration. Kaoutar Elfazazi: conceptualization, validation, writing–review and editing, visualization, supervision, resources, funding acquisition, and project administration.

## Funding

This work was funded by the National Institute of Agricultural Research of Morocco (INRA) under the Medium‐Term Research Program (MTRP 2021‐2025), a fruit‐growing megaproject.

## Ethics Statement

This research did not involve any studies with human participants or animals. All experimental procedures complied with institutional, national, and international ethical standards for scientific research.

## Conflicts of Interest

The authors declare no conflicts of interest.

## Data Availability

All the data relevant to this study have been added within the manuscript.
